# Construction of a density mutant collection in bitter gourd *via* new germplasms innovation and gene functional study

**DOI:** 10.3389/fpls.2022.1069750

**Published:** 2022-11-22

**Authors:** Renbo Yu, Yu Niu, Xiaoyi Wang, Kaili Yang, Xu Han, Zhaohua Liu, Zhiqiang Qi, Yan Yang

**Affiliations:** ^1^Tropical Crops Genetic Resources Institute, Chinese Academy of Tropical Agricultural Sciences, Haikou, Hainan, China; ^2^Hainan Yazhou Bay Seed Lab, Hainan, China; ^3^Key Laboratory of Tropical Crops Germplasm Resources Genetic Improvement and Innovation of Hainan Province, Hainan, China; ^4^Haikou Experimental Station, Chinese Academy of Tropical Agricultural Sciences, Haikou, Hainan, China

**Keywords:** bitter gourd, EMS, mutant collection, phenotype, germplasm

## Abstract

Although a few studies have elucidated the creation of bitter gourd mutants, the suitable concentration and duration of ethyl methanesulfonate (EMS) mutagenesis have not been determined. In this study, mutant collection was conducted to create new germplasms and widen genetic diversity. By employing the seeds of the inbred line Y52 as the mutagenic material, EMS as the mutagen, and the suitable mutagenic conditions for bitter gourd seeds (EMS concentration 0.2%, mutagenic time 10 h), we mutated 10,000 seeds and acquired 3223 independent M_1_ lines. For the randomly selected 1000 M_2_ lines, 199 M_2_ lines with visible phenotypes were found, and 167 M_2_ lines were mutants of fruit shape, size, and tubercles. Furthermore, fourteen dwarf, eleven leaf color, five leaf shape, and eight meristem defect mutants were discovered in this mutant collection. In addition, three lines of 1253, 2284, and 3269 represented recessive mutants crossed with Y52. Furthermore, the yellow leaf lines of 2284 and 3269 were not mutated at the same gene locus. This study constructed a mutant collection through innovative new germplasms and provided valuable resources for bitter gourd breeding and functional gene research.

## Introduction

Bitter gourd (*Momordica charantia* L., 2n = 22), also named balsam pear, bitter melon, bitter cucumber, or African cucumber, belongs to the family *Cucurbitaceae*, and is cultivated in Asia and Africa. As an important vegetable, it has many other pharmacological benefits for human immunodeficiency virus (HIV) infection and diabetes and plays a vital role in our life (Behera et al., 2010). Owing to land shortages and the worsening environment, breeding is focused on promoting crop yield. Bitter gourd is facing the problem of using less land to obtain higher yields, and biotechnology can solve this problem. For a long time, the basic research of bitter gourd was concentrated on establishing recombinant inbred lines and constructing genetic linkage maps using molecular markers (Cui et al., 2017; Cui et al., 2018; Rao et al., 2018; Kaur et al., 2021; Yang et al., 2022; Zhong et al., 2022).

Creating mutant materials to conduct the functional study of related genes in genetic research is necessary when the ratio of spontaneous variation is very low. Consequently, an efficient method for creating mutants is urgently required. Recently, gene editing technology with the CRISPR-Cas system has been widely used in rice, wheat, and tomato (Li et al., 2018; Lin et al., 2020; Wang et al., 2022), but it is coupled with an efficient transformation rate. Nevertheless, the transformation system has not been established in bitter gourd, resulting in the impossibility of gene editing in bitter gourd.

In bitter gourd, the functional study of genetics is considerably behind other *Cucurbitaceae* crops like watermelon and cucumber because of the lack of mutants (Deng et al., 2022; Zhang et al., 2022). A long-recognized large-scale method for mutant acquisition is mutagenization using chemical or physical mutagens. Physical mutagens such as fast neutrons, gamma rays, and x-rays mainly cause nucleotide deletion, and their mutation frequency is low (Li et al., 2017). As a chemical mutagen, EMS mutagenesis was successfully used in crop breeding and functional genomics research. EMS mutagenesis technology is feasible in bitter gourd because it is appropriate to most plants rather than their transformation rate (Lian et al., 2020).

EMS is an effective mutagen that can introduce random base pair changes (mainly G:C/AT) in the genome after replication, bringing less damage to plants (Greene et al., 2003). For example, in *Arabidopsis thaliana*, approximately 700 mutations in each EMS mutant line and 50000 M_1_ lines are sufficient to make a 95% chance of mutation in any G:C base pair in the genome (Jander et al., 2003). Besides, the mutants at M_2_ or more advanced generations of recessive homozygous are crossed with the world-type and generate second filial generation (F_2_) progeny that can be easily discovered by the mutated genes using MutMap (Abe et al., 2012; Qu and Qin, 2014).

Because EMS mutagenesis is easy to control, exhibits a high mutation rate, and causes less harm to plants, it was widely used in rice (Henry et al., 2014), maize (Lu et al., 2018), soybean (Li et al., 2017), pepper (Arisha et al., 2015), cabbage (Sun et al., 2022), watermelon (Deng et al., 2022), cucumber (Zhang et al., 2022) and *Gossypium hirsutum* L (Lian et al., 2020). EMS concentrations differ from one crop to another, and a suitable mutagen concentration can be used to achieve the desired effect. Generally, the optimal lethality among the M_1_ generation is approximately 50% (Arisha et al., 2015; Lian et al., 2020; Dutta et al., 2021). However, the optimal and specific protocol of EMS treatment conditions has not been determined because of limited studies on EMS treatment experiments in bitter gourd. Therefore, there is an urgent need to conduct an EMS mutagenesis study and determine the suitable concentration and duration time of EMS for bitter gourd seeds.

There are few studies on the creation of bitter gourd mutants. Some mutants related to vine length, fertility, and nutrient contents were generated by gamma rays (Co_60_ source) on a small scale (Dutta et al., 2021); however, they did not significantly improve functional genomic research and breeding. Therefore, this study focused on establishing an optimal EMS technique and guidance for other scientists in bitter gourd research. Furthermore, the mutant collection successfully created by EMS mutagenesis will contribute new germplasms to bitter gourd breeding and accelerate the process of gene functional analysis.

## Materials and methods

### Plant material

The cultivated inbred line Y52 was used in this study, and artificial self-pollinated seeds were supplied by the Institute of Tropical Crop Genetic Resources in Hainan province, China.

### EMS experiment

Ten thousand seeds with a small opening at the seed coat of Y52 were soaked in ddH_2_O overnight and then embedded in 0.2% EMS at room temperature with gentle shaking for 10 h. The treated seeds were detoxified with 0.1 mol/L sodium thiosulfate five times. Afterward, the seeds were washed 15 times with ddH_2_O for 5 min each time. Finally, the seeds were wrapped with a wet cloth and transferred to an incubator at 37 °C in a dark condition until germination.

The germinated seeds were planted into small pots 3–5 days later and placed in a greenhouse until 4–6 true leaves appeared. Then, the seedlings were sown into the soil in an open field. By artificial self-pollination, 3223 M_1_ plants generated M_2_ mature seeds, and 10–60 M_2_ seeds were harvested, labeled, and saved. In addition, 10–20 M_2_ seeds from each individual M_1_ plant were randomly selected to sow into the soil, and their morphological phenotype was detected.

## Results

### EMS mutagen experiment condition

Because high concentrations of EMS and prolonged treatment can damage seeds and cause germination problems, we first detected the suitable conditions for bitter gourd mutagenesis. To increase the efficiency of mutagenesis, we created an opening at the embryo of each seed coat. Then, the preliminary assay of EMS treatment was completed, and we counted the germination seeds, and statistical analysis was done 15 days later (**Table 1**).

**Table 1:** The germination rate of different EMS concentration treatment.

**EMS concentration and duration time**	**Total number of seeds**	**The number of germination seeds**	**Germination rate**
0.2% EMS-4h	200	136	68%
	200	141	70.5%
0.2% EMS-10h	200	117	58.5%
	200	106	53%
0.5% EMS-4h	200	80	40%
	200	81	40.5%
0.5% EMS-10h	200	36	18%
	200	33	16.5%
0.1 % Tween20-10h	200	186	93%
	200	193	96.5%

Germination rate: germination seeds / Total number of seeds.

In our preliminary assay, the control group seeds’ germination rate was more than 90%. However, the germination rate with 0.5% EMS treatment in 4 h or 10 h were all lower than 50%, indicating this dose of EMS was not suitable for bitter gourd seeds. In contrast, when the seeds were treated with 0.2% EMS for 10 h, the germination rate was around 50%–60%, which was close to the half-lethal dose, indicating 0.2% EMS for 10 h treatment is the right condition for bitter gourd seeds. Besides, we also used 0.2% EMS for 4 h treatment and found that the mutagenesis effect is poor for bitter gourd.

### M_1_ Population

Y52 is a cultivated species with a length of 30–40 cm and a weight of about 0.5 kg. Therefore, it was selected as the basic material for mutagenesis. Nearly 10,000 seeds were treated with 0.2% EMS for 10 h, and 5319 seeds were germinated, then all the germinated seedlings were planted in the soil (**Table 2**). After one month, we found that 4356 plants survived and 783 plants died, which may have been caused by the injury of the EMS mutagen.

**Table 2:** The germination and survival rate of the M1 generation.

Treatments	0.1 % Tween20	EMS
Total number of seeds	200	10000
Number of germination seeds	191	5319
Number of survival seedlings	186	4536
Fruitful	186	3223
Seed germination rate	95.50%	53.19%
Survival rate	97.38%	85.28%
Fruitful rate	100.0%	71.05%

Germination rate: Number of germination seeds / Total number of seeds, Survival rate: Number of survival seedlings / Number of germination seeds, Fruitful rate: Fruitful / Number of survival seedlings.

### M_2_ Population

Bitter gourd is a monoecious plant with staminate and pistillate flowers born on different internodes. Self-pollination is required for bitter gourd mutant propagation. However, some plants failed to pollinate because female and male flowers did not bloom simultaneously, resulting in no seeds. Finally, only 3223 M_1_ plants were successfully harvested (**Table 2**), and we constructed a bitter gourd mutant collection containing 3223 mutants.

Because bitter gourd needs artificial pollination and has a large workload, we randomly selected 1000 mutants for planting, 10–20 seeds for each, and approximately 10,000–20,000 seedlings were planted. As a result, the phenotypic variation of M_2_ generation was very abundant, approximately 19.9% (**Table 3**), including plant height, leaf color, leaf shape, fruit shape, and meristem defect.

**Table 3:** The mutants of M2 generation.

Mutant phenotype	Number of families	%
Dwarf	14	1.4%
Leaf color	11	1.1%
Leaf shape	5	0.5%
Meristem defect	8	0.8%
Fruit shape	15+152=167	15.8%
In all	205-6=199	19.9%

15+152, “15” indicate 15 lines in Figure 5, “+152” indicate another 152 lines in Supplemental Table 5. “-6” means 6 lines of 681, 1084, 1313, 1372, 1479 and 2078 analyzed twice in this study.

### Dwarf phenotype

**Figure 1:**
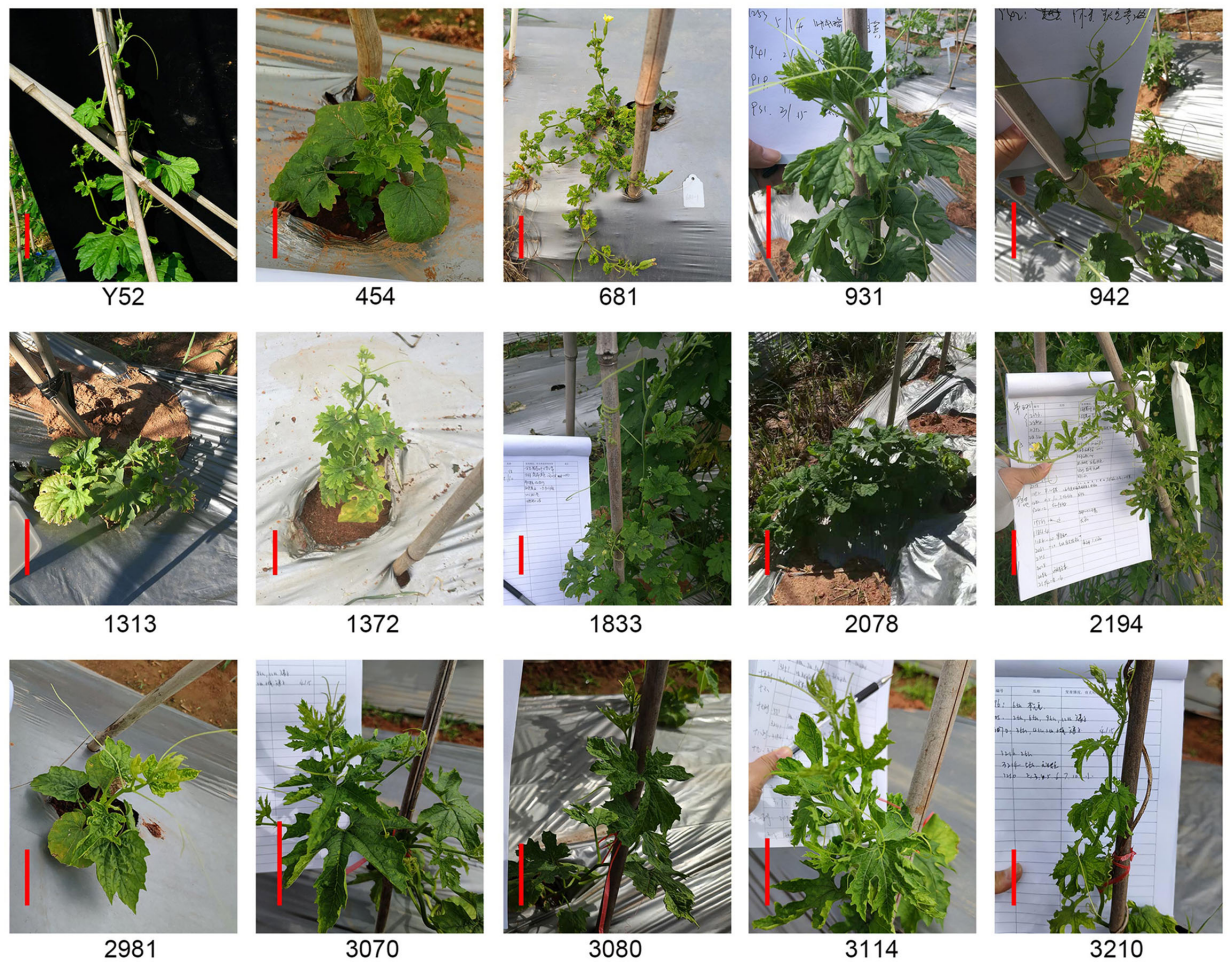
Different dwarf mutants in the M_2_ generation compared to Y52. Scale bar in red color = 5 cm.

Approximately fourteen mutants were not higher than 60 cm or had short internodes, leading to low plant height (**Figure 1**). Among fourteen mutants, nine lines of 454, 931, 1313, 1372, 2078, 2981, 3070, 3080, and 3114 had arrested growth and were no more than 30 cm tall. Moreover, there were 3–4 plants with dwarf phenotype in lines 931, 2981, and 3070 with a segregation rate close to 4:1 (**Supplemental Table 1**), consistent with Mendel’s classical genetic law. In contrast, there were only one or two mutants in other lines, which was inconsistent with Mendelian inheritance law. The possible reason is that the number of family plants is insufficient and does not have a statistical effect. Additionally, lines 681, 942, and 2194 are not only nanoid but also have small leaves, and lines 1833 and 3210 have short internodes (**Figure 1**).

### Leaf color

**Figure 2:**
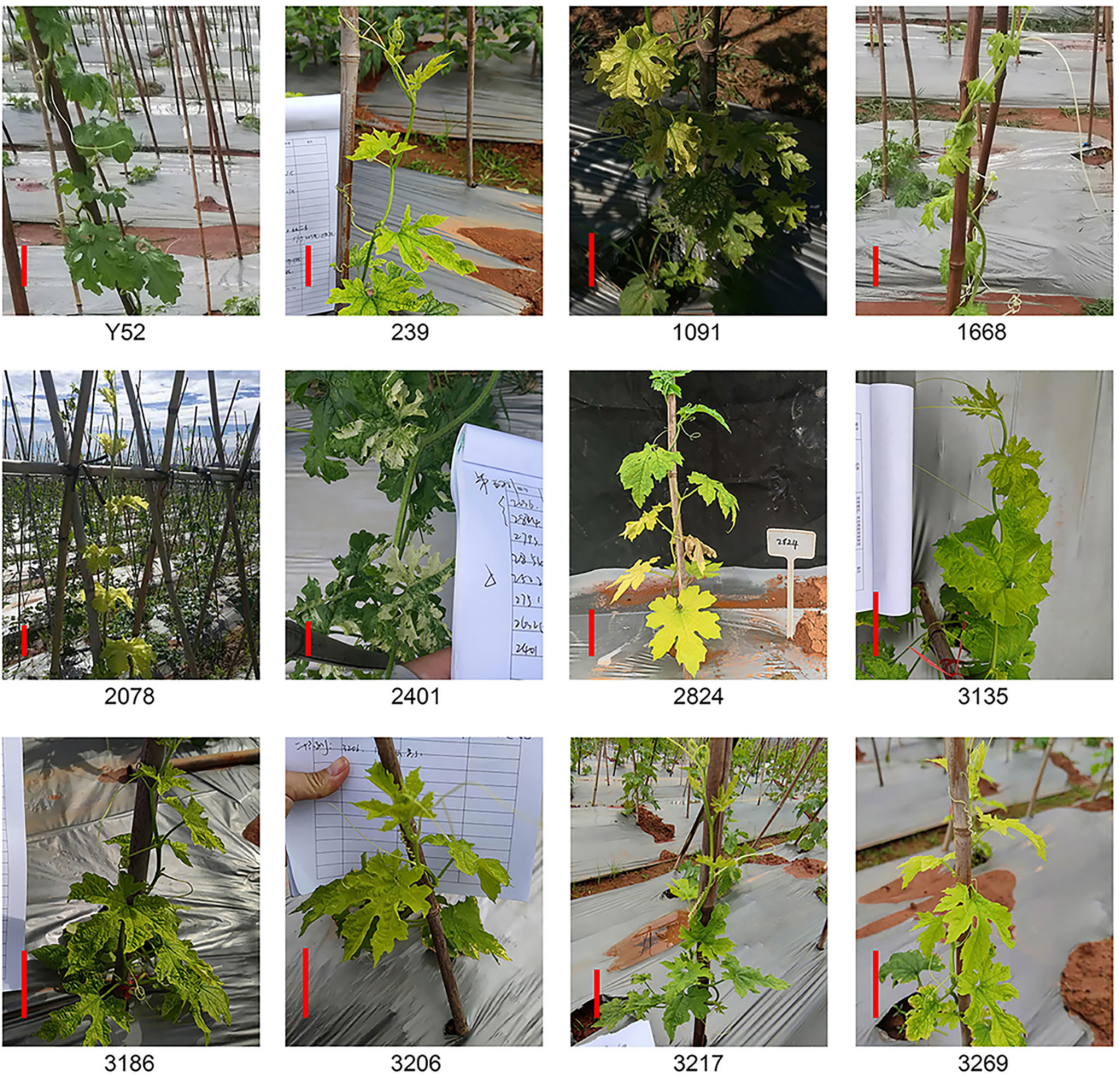
Leaf color mutants of M_2_ plants compared to Y52. Scale bar in red color = 5 cm.

Besides dwarf, leaf color is another easily observed trait. A total of eleven lines had color changes owing to chlorophyll synthesis defects (Abe et al., 2012), mainly leaf yellowing and leaf albinism (**Figure 2**). The leaves of the whole plant line 239, 3135, 3186, and 3206 were light yellow, and five lines of 1091, 1668, 2078, 3217, and 3269 had yellow leaves at the top of the plants while the leaves of line 2824 near the ground were yellow. Furthermore, we also found an albino mutant line 2401 with partial leaves is albinism. Of all these mutant lines, 1668, 2401, 2824, and 3269 had a segregation of 4:1 (**Supplemental Table 2**).

### Leaf shape

**Figure 3:**
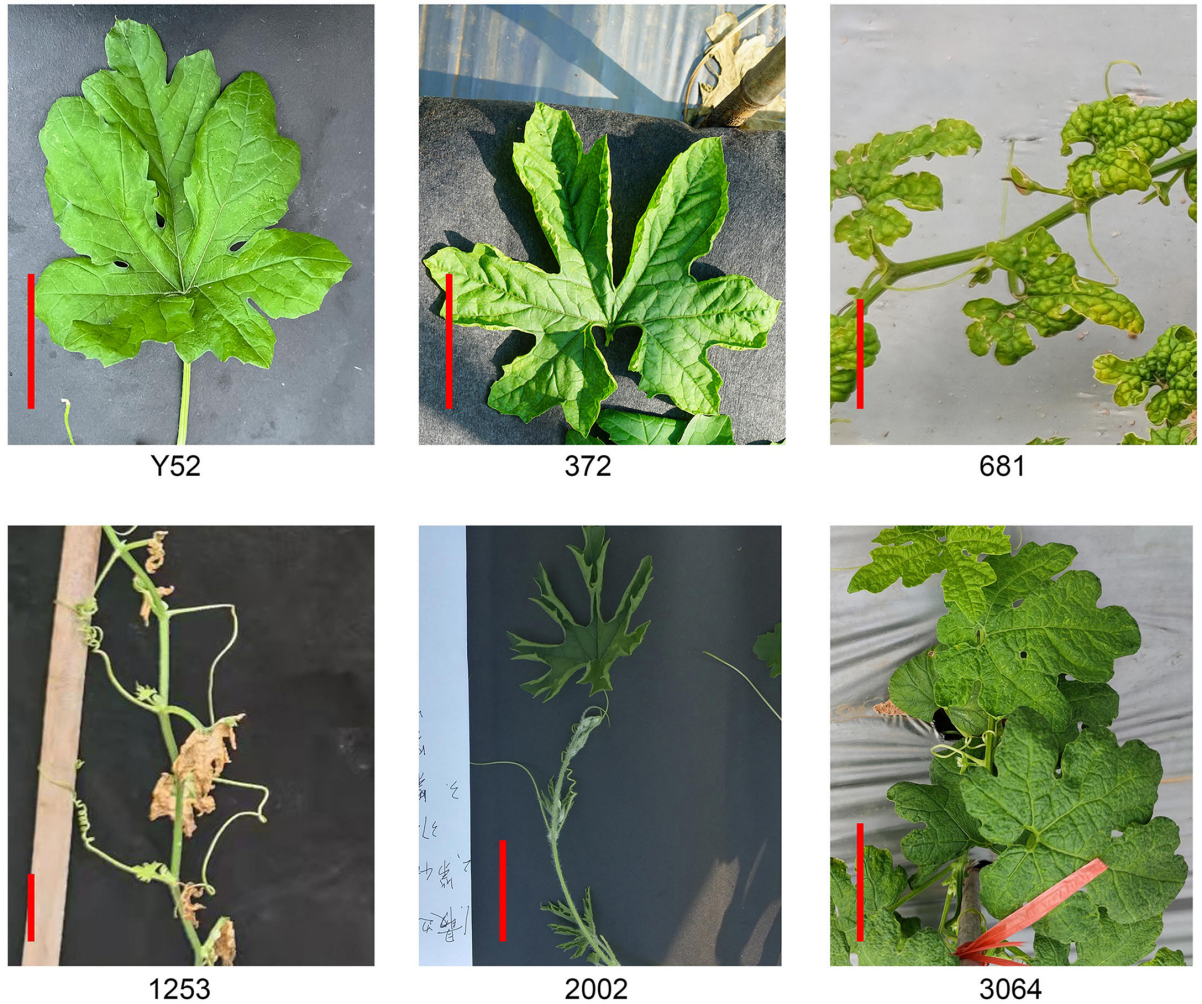
Mutants with a variety of leaf shapes compared to Y52. Scale bar in red color = 5 cm.

We selected five mutants with prominent variations in leaf shape, namely 372, 681, 1253, 2002, and 3064 (**Figure 3**). The leaf blades of lines 372 and 2002 were upward curling, and 2002 is more serious. Line 681 has a small leaf size with some blades bulging upward. Unlike 681, the leaf edge of 3064 is blunt. The mutant of 1253 has severe defect with withered leaves, but they can grow and bloom with fewer female flowers. Unfortunately, the five mutants have the same sterility problem and can only be conservated by heterozygotes which increases the difficulty of gene localization. Moreover, the segregation ratio of 372, 1253, and 3064 are close to 4:1 except the line 681and 2002 (**Supplemental Table 3**).

### Shoot apical meristem defect

**Figure 4:**
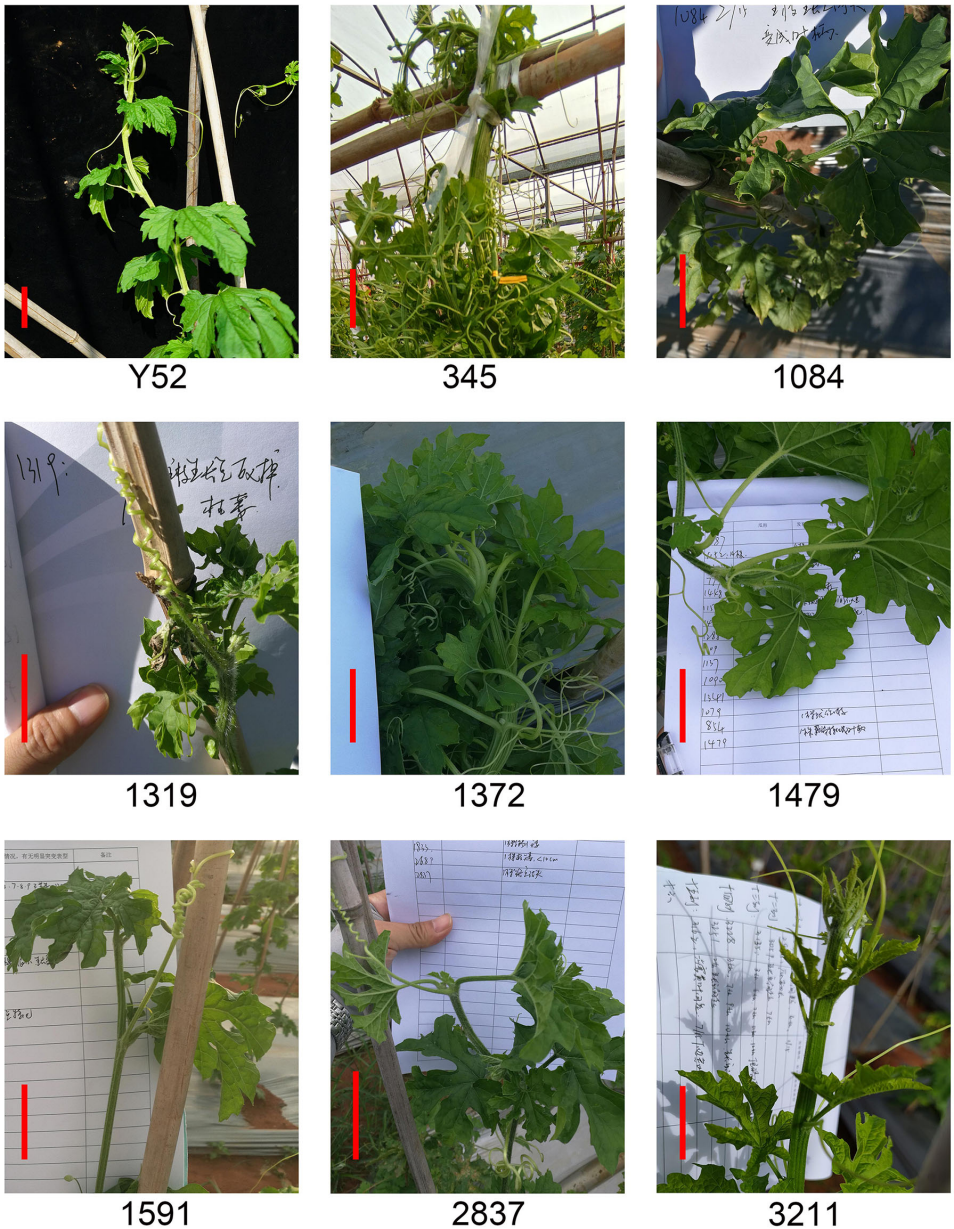
Meristem defect mutants of M_2_ generation compared to Y52. Scale bar in red color = 5 cm.

The shoot apical meristem defect directly affected the yield of bitter gourd. Eight lines with shoot apical meristem defects were obtained by mutant collection screening (**Figure 4**). Three lines of 345, 1372, and 3211 have flat and wide shoot apical meristem, and the apical meristem of lines 1084, 1479, 1519, and 2837 develops into petiole, while the apical meristem of line 1319 was dead or withered. In addition, the segregation of all the lines was inconsistent with the Mendelian genetic law due to insufficient plants (**Supplemental Table 4**).

### Fruit shape mutants

**Figure 5:**
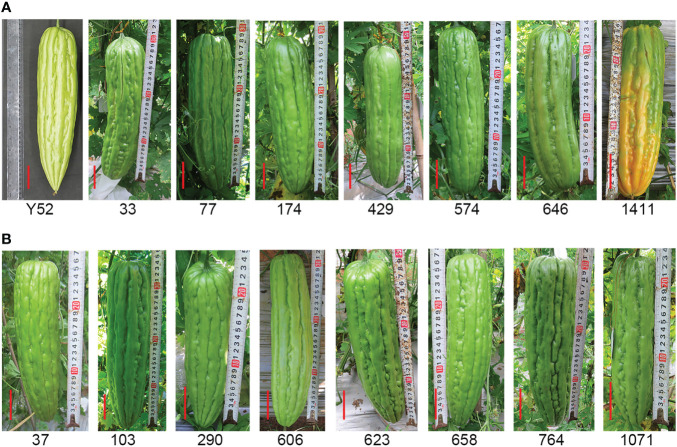
Mutation for fruit shape. **(A)** Mutants with nearly round phenotype at the blossom end. **(B)** Mutants with a truncated phenotype at the base of the fruit compared with Y52. Scale bar in red color = 5 cm.

In our study, the variation of fruit shape was the most abundant phenotype, including fruit length, fruit transverse diameter, the pattern of fruit bibbing and warts, the shape of the blossom end, and the shape of the base of the fruit. From the 1000 M_2_ lines, we screened 167 mutants with various fruit shapes (**Supplemental Table 5**). Among the 167 mutants, eighteen mutants (**Figure 5A**) with a nearly round phenotype at the blossom end and thirty-eight mutants (**Figure 5B**) with a truncated phenotype at the base of the fruit were found compared with Y52.

**Figure 6:**
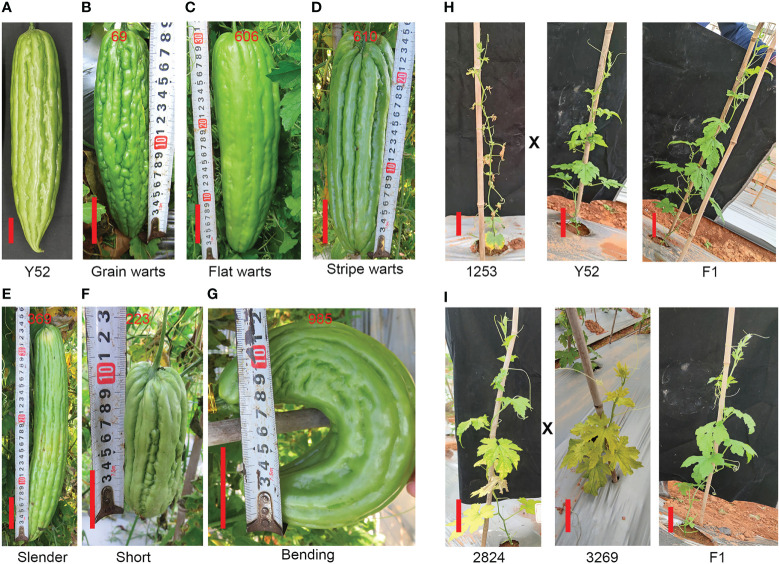
Different kinds of phenotype of fruit **(A–G)** and the phenotype of the F_1_ generation **(H, I)**. **(A)** Fruit of Y52. **(B–D)** The phenotype of fruit with grain, flat and stripe warts. **(E–G)** Slender, short and bending fruit. The phenotype of the F_1_ generation generated by Y52 crossed with 1253 **(H)**, and 2824 crossed with 3269 **(I)** compared to Y52, respectively. Scale bar in red color = 5 cm.

Besides, compared with Y52 (**Figure 6A**) there were three kinds of mutation related to the pattern of fruit bibbing and warts: flat, grain, and stripe warts (**Figures 6B–D**) of three, twenty-eight, and nine mutants, respectively. Second, we found fifteen slender fruit mutants with a small transverse diameter of 3–6 cm (**Figure 6E**). Additionally, there were twenty-six mutants with fruit lengths less than 20 cm, while the fruit length of Y52 was between 30–40 cm (**Figure 6F**). Bending fruit is another prominent phenotype, and twenty-seven mutants were found (**Figure 6G**).

### Mutant analysis

The mutant of 1253 has seventeen plants in the M_2_ generation and five plants with withered leaves, and the segregation ratio was close to 4:1, which means that it is a recessive mutation. To check the recessive or dominant mutation, we hybridized 1253 with Y52 and found that the phenotype of the F_1_ generation was similar to Y52, which indicated that 1253 was a recessive mutant (**Figure 6H**). Furthermore, we selected two mutants of 2824 (segregation ratio 4:1) and 3269 (segregation ratio 4:1) with yellow leaves to establish whether the two mutants mutated at the same gene. We hybridized 2824 with 3269 and found that the phenotype of the F_1_ generation was similar to Y52, indicating that the two mutants had different gene mutations (**Figure 6I**).

### Specific mutants

**Figure 7:**
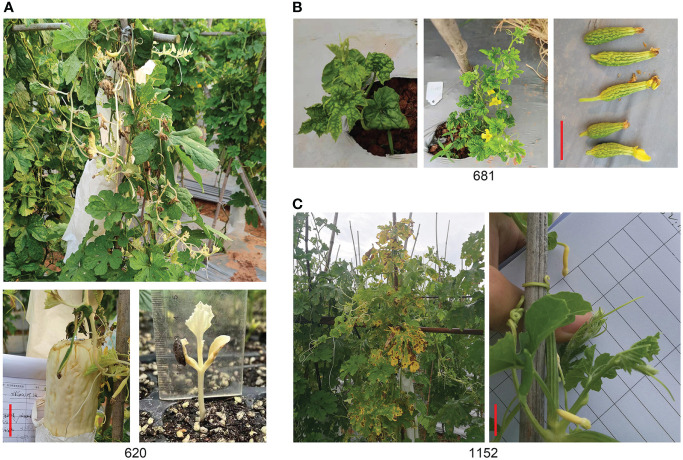
Specific mutants. **(A)** Albino mutant of line 620. **(B)** Thumb fruit of line 681. **(C)** Yellow fruit of line 1152. Scale bar in red color = 5 cm.

Three mutants of 620, 681, and 1152 were selected as specific mutants. Some shoots, leaves, and fruits of 620 were albino, though the seed coat was black; the next generation of seedlings was albino and did not survive (**Figure 7A**). The mutant of 681 has two plants with a dwarf phenotype and thumb-sized fruits without seeds (**Figure 7B**), and it was used as a male parent and hybridized with Y52 to keep it. The mutant of 1152 was another interesting mutant with a yellow ovary and premature senescence of leaves attached to charcoal maggot disease (**Figure 7C**). Unfortunately, the segregation rate of the three mutants was inconsistent with Mendel’s classical genetic law, and the difficulty of gene mapping increased.

## Discussion

Unlike *Arabidopsis thaliana*, rice, wheat, and soybean, the bitter gourd seed covered with a hard coat hindered the full contact between seed and mutagen, leading to decreased mutagenesis efficiency. To solve the problem and improve the mutagenesis efficiency, we used tweezers to clamp a small opening at the seed coat to enable the EMS mutagen to come into full contact with the seeds. Simultaneously, we added 0.1% Tween 20 to enable the seeds to encounter the mutagen evenly and easily clean. In this study, different EMS concentrations and mutagenesis times were evaluated. The germination rate of bitter gourd seeds treated with 0.2% EMS for 10 h was the closest to 50% (Arisha et al., 2015; Lian et al., 2020). Therefore, this condition was selected for subsequent experiments.

Because most of the M_1_ mutants acquired by the EMS mutagenesis were heterozygotes, abundant phenotypic variation was observed in the M_2_ generation. Similar to our results, leaf color mutation was easy to find, and we had already discovered eleven mutants with yellow or yellow-green leaves, and the chlorophyll synthesis deficiency mainly caused the yellow leaf mutants found in previous studies (Abe et al., 2012). Although the leaf color mutants survived but had smaller plants and fruits. Identifying mutation sites that lead to chlorophyll synthesis deficiency is crucial to breeders. Consistent with our results, many gene mutations can cause leaf color deficiency (Ma et al., 2017; Rong et al., 2019), the F_1_ generation produced by 2824 and 3269 did not have yellow leaves (**Figure 6I**), and they have different gene mutations.

Leaf shape, including size, margin, and rolling. Two specific mutants, 372 and 2002, with curly leaves were found in the M_2_ generation. Moderate leaf rolling can help to construct the ideal plant architecture and promote photosynthetic efficiency (Rong et al., 2019). Many genes that control leaf rolling in rice have been cloned, such as *CFL2*, *SRL1*, *ZHD1*, *REL1/2*, and *SLL2* (Zhang et al., 2020). In the family of *Cucurbitaceae*, the *CsPHB* gene was regulated by *miRNA165/166* to control leaf curly in cucumber but was never reported in bitter gourd (Rong et al., 2019). Thus, the two leaf curly mutants were valuable germplasms in bitter gourd and will decipher the gene function of the interest phenotype.

Dwarf mutants are crucial to understanding the regulatory mechanisms for plant height, development, and productivity (Bae et al., 2021) because they can be planted at high density and are resistant to lodging (Zhu et al., 2019; Sun et al., 2020). In addition, a class of plant hormones such as GA, BR, and cytokinin were reported to regulate cell elongation and division that could cause the dwarf phenotype when the level of hormones was abnormal, and many dwarf mutants have been found in various crops such as rice *sd1*, wheat *Rht-B1b/D1b* and cucumber *Csdw* (Wei et al., 2019). Fourteen dwarf mutants with short internodes and small leaf sizes were explored in this study. Previous studies reported that plant height-related genes are associated with leaf sizes such as *OsNAL1*, *OsNAL7*, and *OsDNL-4* (Bae et al., 2021). Similar to recent studies, six mutants of line 681, 942, 1372, 1833, 2194, and 3210 have short plant heights and small leaf sizes.

Fruit phenotype, including fruit shape, size, color, and surface texture possibly caused by long-term domestication, artificial selection, natural selection, or environmental factors, are crucial characteristics for different markets and consumers, and the variety of fruit gives rise to uncovering the molecular mechanism and genetic basis (Snouffer et al., 2020). For example, in bitter gourd, the fruit of wild type with tubercles is small, round, or spindle, while the cultivated species are larger, longer, and do not taper at both ends (Behera et al., 2010). In the mutant collection, we found small, slender, and bending fruits (**Figures 6E–G**). Moreover, we also found some mutants with different tubercles like flat, grain, and stripe warts on the skin (**Figures 6B–D**). Some studies have proved vital genes or QTLs that control fruit shapes, such as *CsFUL* and *CsTRM5* in cucumber, *CmOFP1a* in melon, and *ClSUN25-26-27a* in watermelon (Pan et al., 2020; Snouffer et al., 2020; Boualem et al., 2022). Furthermore, some mutants with a good fruit shape, that do not taper at both ends, and meet our breeding objectives were kept for future study. Therefore, further molecular analysis is needed and important to discover these genes controlling related fruit traits.

## Conclusion

In this study, we confirmed the optimal EMS mutagenesis conditions for bitter gourd seeds of Y52 (EMS concentration 0.2% and mutagenic time 10 h). Furthermore, a mutant collection including 3223 mutants was constructed. We classified them according to the detection of phenotype characters into leaf shape mutation, leaf color mutation, dwarfing mutation, apical meristem mutation, fruit size, and shape mutation. These mutants not only bring new germplasms for breeding but can also accelerate gene functional study in bitter gourd.

## Data availability statement

The original contributions presented in the study are included in the article/**Supplementary Material**. Further inquiries can be directed to the corresponding author.

## Author contributions

RY, YY, and XW conceived and designed the project. RY and XW conducted the EMS mutation experiment, RY and YN collected most of the phenotype. KY, XH, ZL and ZQ participated in some mutant phenotype collected. RY and XW wrote the manuscript, and YY revised the manuscript. All authors contributed to the article and approved the submitted version.

## Funding

This research was supported by grants from the Hainan Province Science and Technology Special Fund (ZDYF2020063), The Major Science and Technology plan of Hainan Province (ZDKJ2021010), Hainan Yazhou Bay Seed Lab (B21HJ0305), and Hainan Province Science and Technology Special Fund (ZDYF2020059).

## Conflict of interest

The authors declare that the research was conducted in the absence of any commercial or financial relationships that could be construed as a potential conflict of interest.

## Publisher’s note

All claims expressed in this article are solely those of the authors and do not necessarily represent those of their affiliated organizations, or those of the publisher, the editors and the reviewers. Any product that may be evaluated in this article, or claim that may be made by its manufacturer, is not guaranteed or endorsed by the publisher.
