# Supplementary material for: Construction of a density mutant collection in bitter gourd via new germplasms innovation and gene functional study
Source: Front Plant Sci. 2022 Nov 22;13:1069750. doi: 10.3389/fpls.2022.1069750 (PMC9724616; doi:10.3389/fpls.2022.1069750)
Supplement: Supplementary file 1 [file Table_1.docx]

| **Supplemental TABLE 1** **\|** The segregation ratio of dwarf mutants. | | | |
| --- | --- | --- | --- |
| **Serial Number** | **Number of lines** | **Mutant phenotype** | **%** |
| 454 | 10 | 1 | 10.0% |
| 681 | 2 | 2 | 100.0% |
| 931 | 15 | 3 | 20.0% |
| 942 | 13 | 1 | 7.69% |
| 1313 | 12 | 1 | 8.33% |
| 1372 | 11 | 2 | 18.2% |
| 1833 | 9 | 4 | 44.4% |
| 2078 | 12 | 2 | 16.7% |
| 2194 | 13 | 2 | 15.4% |
| 2981 | 11 | 3 | 27.3% |
| 3070 | 15 | 4 | 26.7% |
| 3080 | 14 | 2 | 14.3% |
| 3114 | 13 | 2 | 15.4% |
| 3210 | 10 | 7 | 70% |
