# Supplementary material for: Construction of a density mutant collection in bitter gourd via new germplasms innovation and gene functional study
Source: Front Plant Sci. 2022 Nov 22;13:1069750. doi: 10.3389/fpls.2022.1069750 (PMC9724616; doi:10.3389/fpls.2022.1069750)
Supplement: Supplementary file 2 [file Table_2.docx]

| **Supplemental TABLE 2** **\|** The segregation ratio of leaf color mutants. | | | |
| --- | --- | --- | --- |
| **Serial Number** | **Number of lines** | **Mutant phenotype** | **%** |
| 239 | 14 | 7 | 50.0% |
| 1091 | 9 | 1 | 11.1% |
| 1668 | 11 | 3 | 27.3% |
| 2078 | 12 | 2 | 16.7% |
| 2401 | 12 | 3 | 25.0% |
| 2824 | 11 | 3 | 27.3% |
| 3135 | 12 | 5 | 41.7% |
| 3186 | 10 | 1 | 10.0% |
| 3206 | 13 | 2 | 15.4% |
| 3217 | 11 | 1 | 9.1% |
| 3269 | 15 | 4 | 26.7% |
