# Supplementary material for: Construction of a density mutant collection in bitter gourd via new germplasms innovation and gene functional study
Source: Front Plant Sci. 2022 Nov 22;13:1069750. doi: 10.3389/fpls.2022.1069750 (PMC9724616; doi:10.3389/fpls.2022.1069750)
Supplement: Supplementary file 3 [file Table_3.docx]

| **Supplemental TABLE 3 \|** The segregation ratio of leaf shape mutants. | | | |
| --- | --- | --- | --- |
| **Serial Number** | **Number of lines** | **Mutant phenotype** | **%** |
| 372 | 17 | 4 | 23.5% |
| 681 | 2 | 2 | 100.0% |
| 1253 | 17 | 5 | 29.4% |
| 2002 | 14 | 1 | 7.1% |
| 3064 | 20 | 5 | 25.0% |
