# Supplementary material for: Construction of a density mutant collection in bitter gourd via new germplasms innovation and gene functional study
Source: Front Plant Sci. 2022 Nov 22;13:1069750. doi: 10.3389/fpls.2022.1069750 (PMC9724616; doi:10.3389/fpls.2022.1069750)
Supplement: Supplementary file 4 [file Table_4.docx]

| **Supplemental TABLE 4 \|** The segregation ratio of shoot apical meristem defect mutants. | | | |
| --- | --- | --- | --- |
| **Serial Number** | **Number of lines** | **Mutant phenotype** | **%** |
| 345 | 12 | 1 | 8.3% |
| 1084 | 15 | 2 | 13.3% |
| 1319 | 13 | 1 | 7.7% |
| 1372 | 11 | 1 | 9.1% |
| 1479 | 14 | 1 | 7.1% |
| 1591 | 6 | 2 | 33.3% |
| 2837 | 10 | 1 | 10.0% |
| 3211 | 12 | 1 | 8.3% |
