# Supplementary material for: Construction of a density mutant collection in bitter gourd via new germplasms innovation and gene functional study
Source: Front Plant Sci. 2022 Nov 22;13:1069750. doi: 10.3389/fpls.2022.1069750 (PMC9724616; doi:10.3389/fpls.2022.1069750)
Supplement: Supplementary file 5 [file Table_5.docx]

| **Supplemental TABLE 5 \|** The phenotype of fruit mutations. | | |
| --- | --- | --- |
| **Phenotype** | **Serial number** | **Number** |
| Slender | 107, 369, 507, 596, 601, 603, 642, 655, 671, 1153, 1182, 1204, 1310, 1313, 1370 | 15 |
| Short | 17, 74, 123, 197, 218, 222, 247, 270, 277, 295, 298, 331, 453, 582, 633, 718, 87, 967, 973, 991, 996, 1016, 1167, 1349, 1431, 1457 | 26 |
| Nearly round at the blossom end of fruit | 33, 71, 77, 116, 130, 174, 316, 429, 434, 446, 450, 459, 509, 574, 646, 1188, 1249, 1411 | 18 |
| Truncated phenotype at the base of fruit | 30, 37, 66, 98, 103, 112, 133, 159, 169, 172, 192, 211, 237, 290, 323, 329, 414, 416, 448, 512, 522, 545, 571, 604, 611, 623, 658, 764, 927, 1071, 1084, 1109, 1159, 1268, 1308, 1413, 1465, 1770 | 38 |
| Flat warts | 50, 606, 624, 708, 1169, 1229 | 6 |
| Grain warts | 62, 69, 90, 102, 176, 177, 187, 226, 228, 293, 383, 451, 462, 561, 599, 635, 644, 675, 820, 865, 941, 1040, 1183, 1241, 1278, 1354, 1416, 1426 | 28 |
| Stripe warts | 58, 610, 653, 673, 685, 921, 959, 1280, 1341 | 9 |
| Bending fruit | 29, 43, 48, 55, 67, 91, 140, 166, 213, 223, 249, 299, 318, 362, 367, 559, 630, 654, 656, 985, 1137, 1141, 1388, 1389, 1451, 1461, 1479 | 27 |
| In all |  | 167 |
